# *In Vivo* Expression of Reprogramming Factors Increases Hippocampal Neurogenesis and Synaptic Plasticity in Chronic Hypoxic-Ischemic Brain Injury

**DOI:** 10.1155/2016/2580837

**Published:** 2016-11-09

**Authors:** Soohyun Wi, Ji Hea Yu, MinGi Kim, Sung-Rae Cho

**Affiliations:** ^1^Department and Research Institute of Rehabilitation Medicine, Yonsei University College of Medicine, Seoul 03722, Republic of Korea; ^2^Brain Korea 21 PLUS Project for Medical Science, Yonsei University College of Medicine, Seoul 03722, Republic of Korea; ^3^Rehabilitation Institute of Neuromuscular Disease, Yonsei University College of Medicine, Seoul 03722, Republic of Korea; ^4^Yonsei Stem Cell Research Center, Avison Biomedical Research Center, Seoul 03722, Republic of Korea

## Abstract

Neurogenesis and synaptic plasticity can be stimulated* in vivo* in the brain. In this study, we hypothesized that* in vivo* expression of reprogramming factors such as* Klf4*,* Sox2*,* Oct4*, and* c-Myc* would facilitate endogenous neurogenesis and functional recovery. CD-1® mice were induced at 1 week of age by unilaterally carotid artery ligation and exposure to hypoxia. At 6 weeks of age, mice were injected GFP only or both four reprogramming factors and GFP into lateral ventricle. Passive avoidance task and open field test were performed to evaluate neurobehavioral function. Neurogenesis and synaptic activity in the hippocampus were evaluated using immunohistochemistry, qRT-PCR, and/or western blot analyses. Whereas BrdU^+^GFAP^+^ cells in the subgranular zone of the hippocampus were not significantly different, the numbers of BrdU^+^ *β*III-tubulin^+^ and BrdU^+^NeuN^+^ cells were significantly higher in treatment group than control group. Expressions of synaptophysin and PSD-95 were also higher in treatment group than control group. Importantly, passive avoidance task and open field test showed improvement in long-term memory and decreased anxiety in treatment group. In conclusion,* in vivo* expression of reprogramming factors improved behavioral functions in chronic hypoxic-ischemic brain injury. The mechanisms underlying these repair processes included endogenous neurogenesis and synaptic plasticity in the hippocampus.

## 1. Introduction

The strongly established idea that the adult brain has no ability for generating new neurons is now controverted, and recent studies have discovered that the adult mammalian brain can generate new neurons [1, 2]. Even though many studies had a doubt in this idea for a long time, it is now established as the new idea that throughout adult life in many species, including humans, specific regions of the brain continuously generate new neurons [2, 3]. In two regions of the adult brain, adult neurogenesis has been constantly observed. Namely, in the subventricular zone (SVZ) of the lateral ventricle and the subgranular zone of the hippocampus, the adult neurogenesis is generated from neural stem cells (NSCs) [4]. Previous researches in the past decade have clarified procedures of adult neurogenesis, such as the proliferation and the fate determination of NSCs, differentiation and maturation of neurons, and the eventual integration of neurons into the neural networks [2, 5].

The presence of NSCs and the existence of new neurons at certain regions in the adult brain suggest that it may treat incurable neurological diseases by inducing neurogenesis [6]. Previous studies with animal models have also shown that new neural progenitor cells, which have possibility to migrate and effect on damaged regions, are produced at the SVZ [6–8]. Other studies have confirmed that, even at the adult brain, neurogenesis occurred in both the SVZ and subgranular zone of the hippocampus following cerebral ischemia. When the brain has ischemic damage, neuronal precursors of the SVZ and subgranular zone move to the damaged sites [6, 9–11]. Understanding the molecular control of endogenous NSC activation and progenitor cell mobilization will likely give various chances for the use of stimulated neuronal replacement as a therapeutic approach [6, 12]. However, compensatory neurogenesis stimulated after brain injury is basically limited, so it is now obvious that endogenous neurogenesis should not be an individual concern for complete functional recovery [6, 13].

Previous study has been reported that reprogramming factors such as* Oct4*,* Sox2*, c-*Myc*, and* Klf4* can convert fibroblasts into pluripotent stem cells [14]. Enhanced cell proliferation is the most noticeable and earliest response when the reprograming factors are expressed [15]. This enhanced proliferation is associated with the induction of proliferative genes [16]. According to this observation, we suggested that the* in vivo* expression of the four reprogramming factors listed above would support* in vivo* cell proliferation in the hippocampus and SVZ, a major process for recovery from chronic hypoxic-ischemic brain injury.

## 2. Materials and Methods

### 2.1. Animals and Housing

**Figure 1:**
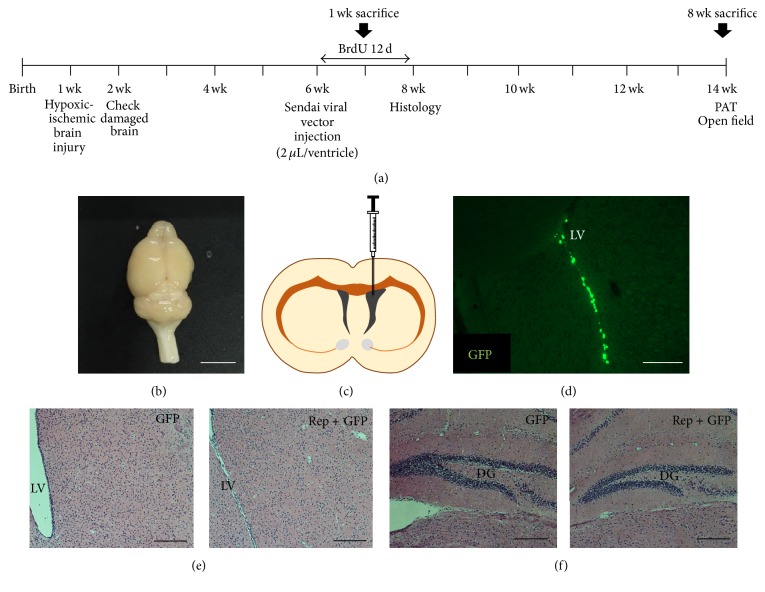
Experimental design and intraventricular injection of reprogramming factors. (a) Schematic timeline of the experiment. (b) A brain picture of chronic hypoxic-ischemic brain injury model. Scale bar = 0.5 cm. (c) At postnatal 6 weeks, mice were injected in the right side of lateral ventricle with GFP and the four reprogramming factors, that is,* Oct4*,* Sox2*, c-*Myc*, and* Klf4* (treatment group), or GFP only (control group). (d) An image of GFP expression in the lateral ventricle. Scale bar = 200 *μ*m. (e) Absence of dysplasia or tumor development in the lateral ventricle. (f) Absence of dysplasia or tumor development in the hippocampus. Both samples of (e) and (f) were evaluated 8 weeks after the treatment, and brain sections were stained with hematoxylin and eosin and observed using a microscope. Neither dysplasia nor tumors were observed in any group. Scale bars = 200 *μ*m. PAT, passive avoidance task; LV, lateral ventricle; DG, dentate gyrus.

All animals were housed in a standard cage (27 × 22.5 × 14 cm^3^) in a facility accredited by the Association for Assessment and Accreditation of Laboratory Animal Care (AAALAC) and provided food and water* ad libitum* with alternating 12-hour light/dark cycles, according to animal protection regulations. The experimental procedures were approved by the Institutional Animal Care and Use Committee (IACUC number 2016-0109 and number 2016-0070). A schematic timeline of this experiment from birth to 14 weeks of age is provided in Figure 1(a).

### 2.2. Neonatal Hypoxic-Ischemic Brain Injury

Permanent ischemic brain damage was induced in 7-day-old CD-1 (ICR) mice (Orient, Seongnam, Korea), by unilateral right carotid artery ligation. Hypoxic brain injury (8% O_2_ for 60 minutes) was also induced as described previously [17–20]. Animals with severe brain lesions covering more than 50% of the unilateral hemisphere were excluded from the criteria. Atmosphere around mice was maintained at 37°C within the hypoxic chamber. Neonatal hypoxic-ischemic brain injury model is shown in Figure 1(b).

### 2.3. Intraventricular Injection of Reprogramming Factors

At 6 weeks of age, a total of 32 mice were anesthetized with ketamine (100 mg/kg; Huons, Gyeonggi-do, Korea) and xylazine (10 mg/kg; Bayer Korea, Seoul, Korea) by intraperitoneal (IP) injection and were randomly assigned to either the control group (green fluorescent protein (GFP) only, *n* = 15) or the treatment group (*Oct4*,* Sox2*, c-*Myc*, and* Klf4*, and GFP, *n* = 15). Mice received intraventricular injection of GFP (1.5 × 10^6^ CIU/mL, 2 *μ*L volume, and 0.01 *μ*L/s infusion rate; ThermoFisher, Carlsbad, CA, USA) only or both GFP and four reprogramming factors (1.5 × 10^6^ CIU/mL, 2 *μ*L volume, and 0.01 *μ*L/s infusion rate; CytoTune™-iPS 2.0 Sendai Reprogramming Kit, ThermoFisher, Carlsbad, CA) using stereotaxic coordinates (AP +0.5 mm from bregma; ML −0.7 mm from bregma; DV −2.0 mm from dura) (Figure 1(c)). Mice had recovered in a heating chamber at 37°C after the surgical treatment.

### 2.4. Passive Avoidance Task

To evaluate memory function based on learning to avoid an aversive stimulus, a 2-compartment step-through passive avoidance task (PAT) was conducted [21–23]. The method was adapted from the previous study for examination of the long-term memory [23].

### 2.5. Open Field Test

Open field test is generally used to evaluate locomotor activity and spontaneous exploration in a novel environment [24, 25]. Activity monitoring was conducted in a square area measuring 30 × 30.5 × 31 cm^3^. The area's floor was divided into 16 sectors. The 4 inner sectors marked out the center, while the 12 outer sectors were defined as the periphery. Total distance in the center was recorded as an index of anxiety [26, 27]. Mice were placed individually into the periphery of the area and were allowed to explore freely for 15 minutes while being monitored with a video camera. The resulting data were analyzed using the video tracking system Smart Vision 2.5.21 (Panlab, Barcelona, Spain).

### 2.6. Immunohistochemistry

This method was adapted from the previous study [17]. Immunohistochemistry was performed as described previously [17]. Briefly, animals were euthanized and perfused with 4% paraformaldehyde (PFA). Harvested brain tissues were cryosectioned with a slice thickness of 16 *μ*m and immunohistochemistry staining was performed on 4 sections. All animals received an IP injection of 5-bromo-2-deoxyuridine (BrdU) (50 mg/kg) once a day for 12 days, beginning 1 day after stereotaxic surgery, to evaluate endogenous cell genesis and neurogenesis in the subgranular zone of the hippocampus and SVZ. Eight weeks after the treatment, the long-term survival of newly generated neurons was evaluated in 3 mice from each of the control and treatment groups (*n* = 3 per group). Sections were stained with primary antibodies against BrdU (1 : 200, Abcam, Cambridge, UK), *β*III-tubulin (1 : 400, Covance, Princeton, NJ, USA), glial fibrillary acidic protein (GFAP) (1 : 400, Abcam), Nestin (1 : 400, Abcam), synaptophysin (1 : 100, Abcam), and postsynaptic density protein 95 (PSD-95) (1 : 100, Abcam) and secondary antibodies such as Alexa Fluor® 488 goat anti-rat (1 : 400, Invitrogen, Carlsbad, CA, USA), Alexa Fluor 568 goat anti-rabbit (1 : 400, Invitrogen), and Alexa Fluor 594 goat anti-mouse (1 : 400, Invitrogen). Stained sections were then mounted on glass slides with fluorescent mounting medium containing 4′,6-diamidino-2-phenylindole (DAPI; Vectashield, Vector, Burlingame, CA, USA). Stained sections were analyzed using confocal microscopy (LSM700, Zeiss, Gottingen, Germany). Images of GFP expression in the lateral ventricle were taken using a fluorescent microscope (Axio Imager M2, Zeiss) and density was evaluated using ZEN Imaging Software (blue edition, Zeiss).

### 2.7. Western Blot Analysis

This method was adapted from the previous study [17]. To confirm the expression of synaptophysin and PSD-95 in the hippocampus in the control and treatment groups (*n* = 3 per group), 50 *μ*g extracted proteins were dissolved in sample buffer (60 mM Tris–HCl, pH 6.8, 14.4 mM b-mercaptoethanol, 25% glycerol, 2% SDS, and 0.1% bromophenol blue; Invitrogen), incubated for 10 minutes at 70°C, and separated on a 10% SDS reducing polyacrylamide gel (Invitrogen). Separated proteins were then equally loaded and transferred onto polyvinylidene difluoride membranes (Invitrogen) using a Trans-Blot System (Novex® Mini-Cell; Invitrogen). Blots were blocked for 1 hour in Tris-buffered saline (TBS) (10 mM Tris-HCl, pH 7.5, and 150 mM NaCl) containing 5% nonfat dry milk (Bio-Rad) at room temperature, washed three times with TBS, and incubated at 4°C overnight with a synaptophysin (1 : 1,000, Abcam) antibody and a PSD-95 (1 : 1,000, Abcam) antibody in TBST (10 mM Tris, pH 7.5, 150 mM NaCl, and 0.02% Tween 20) containing 3% nonfat dry milk. The next day, blots were washed three times with TBST and incubated for 1 hour with horseradish peroxidase-conjugated secondary antibodies (1 : 3,000, Santa Cruz Biotechnology, Santa Cruz, CA, USA) at room temperature. A housekeeping gene was evaluated with actin-antibody (1 : 1,000, Santa Cruz Biotechnology). After washing three times with TBST, blots were visualized with an ECL detection system (Amersham Pharmacia Biotech, Little Chalfont, UK). Using ImageQuant™ LAS 4000 software (GE Healthcare Life Science, Chicago, IL, USA), western blot results were saved into TIFF image files, and then the images were analyzed using Multi-Gauge (Fuji Photo Film, version 3.0, Tokyo, Japan).

### 2.8. Quantitative Real-Time Reverse Transcription-Polymerase Chain Reaction (qRT-PCR)

cDNAs were synthesized from sample RNAs with ReverTra Ace® qPCR RT Master Mix with gDNA Remover (TOYOBO, Osaka, Japan). Then, 5 *μ*L of cDNA in a total volume of 20 *μ*L was used in the following reaction. The following steps of qRT-PCR are the same as the previous study [28]. The primers were as follows: mouse* PSD-95*, 5′-TCCCCATTTTCTCCCACACAC-3′ and 5′-ACGGCGTGGGGAGTTATGAT-3′; mouse* synaptophysin*, 5′-GTGCCAACAAGACGGAGAGT-3′ and 5′-CACCCGAGGAGGAGTAGTCA-3′; mouse glyceraldehyde 3-phosphate dehydrogenase (*GAPDH*), 5′-CATCACTGCCACCCAGAAGACTG-3′ and 5′-ATGCCAGTGAGCTTCCCGTTCAG-3′.* GAPDH* was used as the internal control.

### 2.9. Statistical Analyses

The numbers of BrdU^+^ cells, BrdU^+^ *β*III tubulin^+^ cells, BrdU^+^NeuN^+^ cells, and BrdU^+^GFAP^+^ cells in the hippocampus, the level of synaptic proteins such as synaptophysin and PSD-95, and open field test results were analyzed between the treatment and control groups using independent *t*-test as implemented in SPSS (version 18.0; Armonk, NY, USA). Neurobehavioral outcome for passive avoidance task was compared between baseline and 24 hours using paired *t*-test. Statistical significance was accepted when *p* < 0.05.

## 3. Results

### 3.1. *In Vivo* Reprogramming Factor Expression Improves Long-Term Memory after Chronic Hypoxic-Ischemic Brain Injury

**Figure 2:**
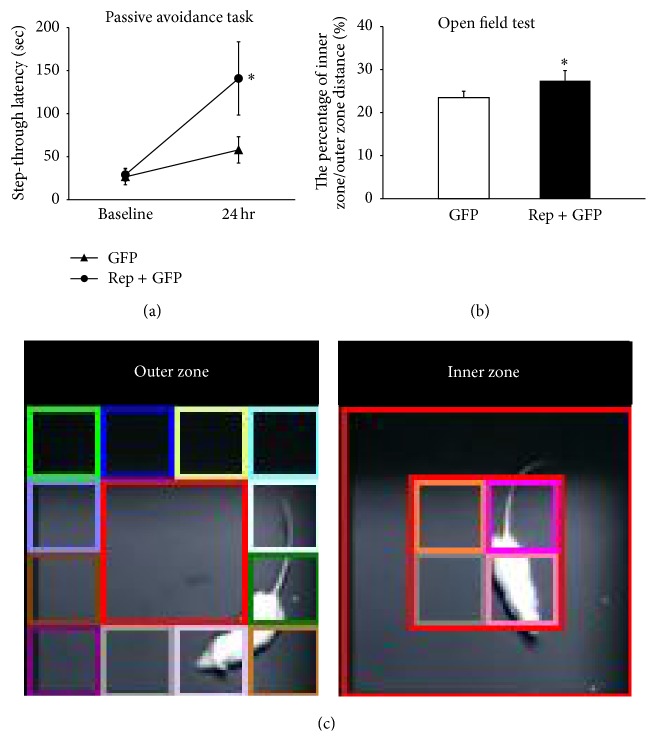
Effects of reprogramming factor expression on neurobehavioral functions after chronic hypoxic-ischemic brain injury. Passive avoidance task and open field test results showed that expression of the four reprogramming factors improved long-term memory and decreased anxiety 8 weeks after the treatment. (a) Expression of the four reprogramming factors significantly improved retention test performance 24 hours after an aversive stimulus 8 weeks after the treatment (140.9 ± 42.5 seconds) relative to that in the initial assessment (57.9 ± 15.3 seconds), whereas the performance was not statistically improved in the control group (*t* = 2.916, *p* < 0.05; *n* = 7 per group). The result suggests that the expression of reprogramming factors improves long-term memory 8 weeks after the treatment. (b) The total distance of movement was assessed for 15 min. The percentage of the inner zone/outer zone increased in the treatment group (27.4 ± 1.3%) compared to the control group (23.5 ± 1.1%) (*t* = 2.568, *p* < 0.05; *n* = 8 and *n* = 9 per group, resp.). (c) Among the total of 16 zones, 4 central zones are an inner zone, and 12 peripheral zones are an outer zone. ^*∗*^ *p* < 0.05.

To determine whether the* in vivo* expression of four reprogramming factors improved cognitive function, passive avoidance task was performed before the surgical treatment and 8 weeks after intervention in the control and treatment groups (*n* = 7 per group). Expression of the reprogramming factors significantly improved retention test performance 24 hours after an aversive stimulus at 8 weeks after the treatment (140.9 ± 42.5 seconds) relative to that in the initial assessment (57.9 ± 15.3 seconds) (*t* = 2.916, *p* < 0.05), whereas the performance was not statistically improved in the control group (Figure 2(a)). The result suggests that* in vivo* expression of reprogramming factors improves long-term memory 8 weeks after the treatment. Additionally, when the step-through latency was evaluated in the passive avoidance task, there was no significant difference between sham-operated control group (205.7 ± 55.7 seconds) and treatment group (235.7 ± 45.7 seconds) (*n* = 6 per group; Figure S2A in Supplementary Material available online at http://dx.doi.org/10.1155/2016/2580837).

### 3.2. *In Vivo* Expression of Reprogramming Factors Decreases Anxiety in Chronic Hypoxic-Ischemic Brain Injury

To evaluate the effect of the four reprogramming factors on anxiety, the results of open field test, 8 weeks after intervention, were compared between the treatment and control groups (*n* = 8 and 9 per group, resp.). The total zone was divided into the outer zone and inner zone (Figure 2(c)). The percentage of the inner zone/outer zone increased after the* in vivo* expression of the four reprogramming factors in the treatment group (27.4 ± 1.3%) compared to the control group (23.5 ± 1.1%) (*t* = 2.568, *p* < 0.05; Figure 2(b)). When a ratio of distance of inner zone over outer zone decreases, this ratio can be the indication of the reduction of anxiety [27]. Therefore, this result suggests that the* in vivo* expression of reprogramming factors decreased anxiety-related behavior. Additionally, when the percentage of the inner zone/outer zone was evaluated in sham-operated control group (9.1 ± 2.0%) and treatment group (10.3 ± 3.3%), there was no significant difference between two groups (*n* = 6 per group; Figure S2B).

### 3.3. *In Vivo* Reprogramming Factor Expression Increases the Number of New Neurons but Not Astrocytes in the Hippocampus

**Figure 3:**
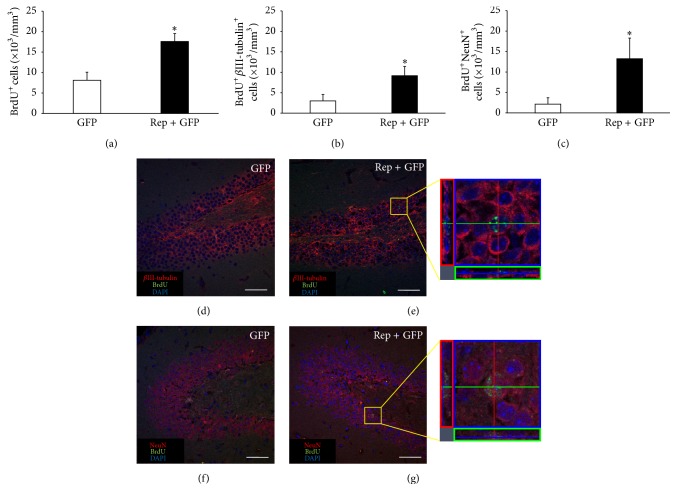
The number of new neurons in the hippocampus increased by* in vivo* expression of reprogramming factors. At postnatal 6 weeks, mice were injected with viral vector expressed GFP only (control group) or the four reprogramming factors and GFP (treatment group). To identify newly generated cells, mice were injected daily with 5-bromo-2-deoxyuridine (BrdU) up to 12 days. Eight weeks after injection, histological evaluations were performed. (a) The density of BrdU^+^ cells in the hippocampus was significantly higher in the treatment group than in the control group (*t* = 3.528, *p* < 0.05). (b-c) The density of newly generated neurons was determined through confocal microscopy by calculating the density of cells triple positive for DAPI (blue, nuclei), BrdU (green), and cell type-specific markers such as *β*III-tubulin and NeuN. The densities of BrdU^+^ *β*III-tubulin^+^ (b) and BrdU^+^NeuN^+^ (c) cells were significantly higher in the treatment group than the control group (*t* = 2.450, *p* < 0.05 and *t* = 2.297, *p* < 0.05, resp.). (d–g) Confocal microscope images are immunohistochemistry results. (e, g) Cells with triple positive for DAPI, BrdU, and cell type-specific markers are indicated in the yellow box at the right panel. Scale bars = 50 *μ*m. ^*∗*^ *p* < 0.05.

To determine the density of proliferating cells in the subgranular zone of the hippocampus, BrdU^+^ cells were counted. The number of BrdU^+^ cells in the treatment group (17.6 ± 1.9 × 10^3^ cells) was significantly 2.2 times higher than in the control group (8.1 ± 2.0 × 10^3^ cells) (*t* = 3.528, *p* < 0.05; Figure 3(a)). Meanwhile, to evaluate the cell fate of proliferating cells in the hippocampus, double-staining of cells with BrdU and cell type-specific markers such as *β*III-tubulin (Tuj1, early neuronal marker), NeuN (mature neuronal maker), or GFAP (astrocyte marker) was performed. The numbers of BrdU^+^ *β*III-tubulin^+^ cells in the treatment group (9.2 ± 2.3 × 10^3^ cells) were significantly 3.1 times higher than in the control group (3.0 ± 1.6 × 10^3^ cells) (*t* = 2.450, *p* < 0.05; Figures 3(b), 3(d), and 3(e)), indicating that the* in vivo* expression of reprogramming factors enhanced neurogenesis. The number of BrdU^+^NeuN^+^ cells in the treatment group (13.2 ± 5.0 × 10^3^ cells) was also significantly 6.2 times higher than in the control group (2.1 ± 1.5 × 10^3^ cells) (*t* = 2.297, *p* < 0.05; Figures 3(c), 3(f), and 3(g)), suggesting that the newly generated neurons differentiate into mature neurons. However, there were no BrdU^+^GFAP^+^ cells in the hippocampus in either group suggesting that neurogenesis in the hippocampus is towards neurons, not astrocytes. The number of BrdU^+^ cells of two weeks after the surgical treatment groups is shown in Figure S1. The above values are described in Table 1.

### 3.4. *In Vivo* Expression of Reprogramming Factors Increases the Number of Neural Precursor Cells in the Subventricular Zone

**Figure 4:**
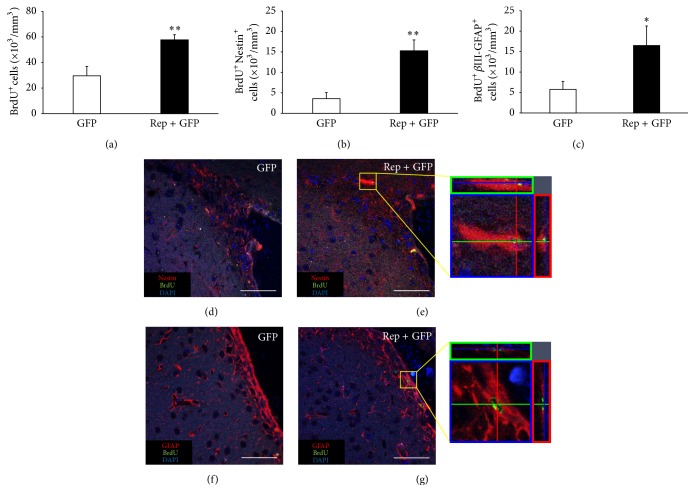
The number of neural progenitor cells in the subventricular zone increased by the* in vivo* expression of reprogramming factors. (a) The density of BrdU^+^ cells in the subventricular zone was significantly higher in the treatment group than the control group (*t* = 3.596, *p* < 0.01). (b-c) The density of neural progenitor cells was determined through confocal microscopy by calculating the density of cells triple positive for DAPI (blue, nuclei), BrdU (green), and cell type-specific markers such as Nestin and GFAP. The densities of BrdU^+^Nestin^+^ cells (b) and BrdU^+^GFAP^+^ cells (c) were significantly higher in the treatment group than the control group (*t* = 3.868, *p* < 0.01 and *t* = 2.631, *p* < 0.05, resp.). (d–g) Confocal microscope images are immunohistochemistry results. (e, g) Cells with triple positive for DAPI, BrdU, and cell type-specific markers are indicated in the yellow box at the right panel. Scale bars = 50 *μ*m. ^*∗*^ *p* < 0.05; ^*∗∗*^ *p* < 0.01.

To determine the density of proliferating cells in the SVZ of the lateral ventricle, BrdU^+^ cells were counted. The number of BrdU^+^ cells in the treatment group (57.7 ± 4.1 × 10^3^ cells) was 2.0 times higher than in the control group (29.6 ± 7.4 × 10^3^ cells) (*t* = 3.596, *p* < 0.01; Figure 4(a)). Meanwhile, to evaluate the proliferating cells in the SVZ, double-staining of cells with BrdU and cell type-specific markers such as Nestin (neural progenitor marker) and GFAP was used. The numbers of BrdU^+^Nestin^+^ cells in the treatment group (15.3 ± 2.6 × 10^3^ cells) were 4.3 times higher than in the control group (3.6 ± 1.5 × 10^3^ cells) (*t* = 3.868, *p* < 0.01; Figures 4(b), 4(d), and 4(e)), indicating that the* in vivo* expression of reprogramming factors increased newly generated neural precursor cells. The number of BrdU^+^GFAP^+^ cells in the treatment group (16.5 ± 4.8 × 10^3^ cells) was also 2.9 times higher than in the control group (5.7 ± 2.0 × 10^3^ cells) (*t* = 2.631, *p* < 0.05; Figures 4(c), 4(f), and 4(g)), suggesting that the* in vivo* expression of reprogramming factors increased neural precursor cells and/or immature astrocytes in the SVZ. The above results are described in Table 1.

### 3.5. *In Vivo* Expression of Reprogramming Factors Increases Synapse Density in the Hippocampus

**Figure 5:**
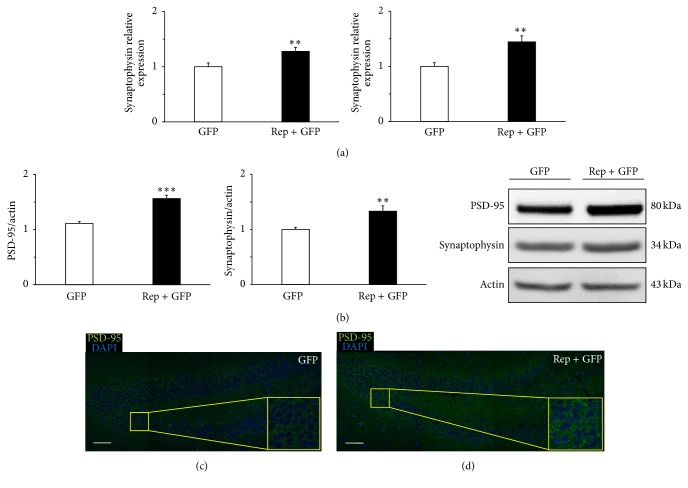
Synaptic density in the hippocampus increased by* in vivo* expression of reprogramming factors. The expression levels of the synaptic markers of PSD-95 and synaptophysin in the hippocampus were determined by quantitative real-time reverse transcription-polymerase chain reaction (qRT-PCR) and western blot (*n* = 3 per group). (a) qRT-PCR analysis confirmed that PSD-95 and synaptophysin levels were significantly higher in the treatment group than the control group (*t* = 2.879, *p* < 0.01; *t* = 3.668, *p* < 0.01, resp.). (b) Western blot analysis also confirmed that PSD-95 and synaptophysin levels were significantly higher in the treatment group than the control group (*t* = 6.499, *p* < 0.001; *t* = 3.136, *p* < 0.01, resp.). (c, d) Confocal microscope images are immunohistochemistry results that showed the area of the dentate gyrus in the hippocampus. The expression of PSD-95 in the yellow box represented the difference of synaptic density in the control (c) and treatment (d) group. Scale bars = 50 *μ*m. ^*∗∗*^ *p* < 0.01; ^*∗∗∗*^ *p* < 0.001.

We evaluated expression levels of a presynaptic maker (synaptophysin) and postsynaptic marker (PSD-95) to determine if reprogramming factor expression increased density of synapse in the hippocampus. Using qRT-PCR, we confirmed that the level of PSD-95 was higher in the treatment group than the control group (1.3 ± 0.1-fold, *t* = 2.879, and *p* < 0.01; Figure 5(a)). We also confirmed that the level of synaptophysin was higher in the treatment group than the control group (1.4 ± 0.1-fold, *t* = 3.668, and *p* < 0.01; Figure 5(a)). Using western blot analysis, we confirmed that the level of PSD-95 was higher in the treatment group than the control group (1.6 ± 0.1-fold, *t* = 6.499, and *p* < 0.001; Figure 5(b)). We also confirmed that the level of synaptophysin was higher in the treatment group than the control group (1.3 ± 0.1-fold, *t* = 3.136, and *p* < 0.01; Figure 5(b)). Confocal images also showed that PSD-95 expression was higher in the treatment group (Figures 5(c) and 5(d)). These results suggest that* in vivo* reprogramming therapy increases hippocampal synaptic plasticity in chronic hypoxic-ischemic brain injury.

## 4. Discussion

In our study, the reprogramming factors such as* Oct4*,* Sox2*, c-*Myc*, and* Klf4* were delivered by intraventricular injection of viral vector, and expression of GFP was confirmed in the lateral ventricle near the dentate gyrus. Nakatomi et al. [29] reported that, following ischemic brain injury, adult neural progenitors can be encouraged* in situ* by intraventricular infusion of growth factors to substitute CA1 pyramidal neurons in the hippocampus. Intraventricular infusion of growth factors recruited the endogenous progenitor* in situ*, so making huge regeneration of pyramidal neurons after ischemia because hippocampal CA1 pyramidal neurons undertake general degeneration following temporary ischemia [30–32]. Using a similar appoach, activated neuronal progenitor cells in the subgranular zone of the dentate gyrus migrate and differentiate into new neurons [33], and subependymal progenitors of the lateral ventricular wall might increase the movement to the pools of neurogenic progenitors in the hippocampus of the mammalian brain [12, 34, 35]. Taken together, these results suggest that hippocampal neurogenesis might be increased by delivering reprogramming factors into the lateral ventricle.

Histological analysis in the hippocampus showed that the* in vivo* expression of reprogramming factors enhanced the proliferative generation of neurons, but not astrocytes. In a previous study, when double-labeling was performed, approximately 60% of BrdU^+^ cells in the granule cell layer were also NeuN double-stained, indicating differentiation into neurons. None of the BrdU^+^ cells in the granule cell layer appeared to differentiate into GFAP^+^ astrocytes [36]. However, it cannot be ruled out that these cells could be also derived from other cell types because* in vivo* overexpression of reprogramming factors can epigenetically activate cells so that they are in an intermediate plastic state that allows them to have an alternative fate [37–41]. Tentatively, our study demonstrated that four reprogramming factors may influence endogenous progenitors in the hippocampus to differentiate into mature neurons.

In addition, histological analysis in the SVZ showed that the* in vivo* expression of reprogramming factors increased the neural precursor cells. In the SVZ, stem cell astrocyte marker (type B cells, glial fibrillary acidic protein expressing (GFAP)) also shared neural stem cell marker (Nestin) [42, 43]. Thus, the GFAP^+^ cells generated by reprogramming factor expression might be a novel cell source with stem cell potential in the SVZ of the injured brain [28, 43].

For the learning of new memories, hippocampal neurogenesis may be essential [44]. Both learning and the affective state are related to the alteration of adult hippocampal neurogenesis [45]. In previous studies, genetic ablation of new neurons in the hippocampus was associated with learning and memory impairment, while induction of hippocampal neurogenesis alleviated such lesion-induced impairment [46, 47]. Therefore, new neurons in hippocampus play an important role in the hippocampus-related behaviors such as learning and memory [48]. In particular, an increase in neurogenesis, and thus an increase in neural plasticity, may improve performance in learning and memory tasks [44]. Conversely, blocking neurogenesis may be the cause of the observable decline in performance in various learning- and memory-related tasks [44].

In our study, a ratio of distance in the center and periphery was recorded as an index of anxiety. When a mouse is placed in a foreign environment, it intends to stay in the outer zone. After it adapts in the environment and its anxiety decreases, the spending time and distance increase in the inner zone. Therefore, increase of spending time or distance in the central part and of the ratio central over total locomotion indicates decrease of anxiety [27]. Several researchers have suggested a link between hippocampal neurogenesis and anxiety-related behaviors. Revest et al. convincingly explained the connection between neurogenesis and anxiety-related behavior [49]. In that study, the authors demonstrated that deficits in hippocampal neurogenesis via specific ablation of newborn neurons in the adult dentate gyrus resulted in anxious behavior [50]. Furthermore, Dias and colleagues also found a decreased number of immature neurons in the dentate gyrus of a rodent model with generalized anxiety [51]. Consistent with the previous studies [44–51], we confirmed that the* in vivo* expression of reprogramming factors facilitated hippocampal neurogenesis and hippocampus-related behavioral outcomes such as increased long-term memory and decreased anxiety-related behavior. Additionally, when neurobehavioral tests were performed in sham-operated control group and treatment group, there was no significant difference between two groups. Therefore, it showed that the* in vivo* expression of reprogramming factors might be effective in the microenvironment of the injured brain.

Our qRT-PCR, western blotting, and immunohistochemistry results showed that* in vivo* reprogramming therapy increased expression of synaptic proteins such as the presynaptic marker, synaptophysin, and postsynaptic marker, PSD-95, relative to the control group. PSD-95 is a core component of the PSD and a key molecule in mature synapses [52]. Moreover, maturation of synapse—presynaptic terminal and postsynaptic component—can be labeled by PSD-95 [53]. In previous studies, targeted disruption of PSD-95 alters activity-dependent synaptic plasticity and learning. The reason of why PSD-95 mutant mice are impaired in spatial learning can be explained by these abnormalities in synaptic plasticity [54, 55]. In addition, PSD-95 mutant mice exhibit severe deficits in spatial, working, and distress memory and abnormal anxiety, and behaviors are likely to be related to abnormal synaptic transmission in the hippocampus, especially dentate gyrus synapses [52]. Here we confirmed that increased expression of PSD-95 and synaptophysin was related to improved long-term memory and decreased anxiety due to enhanced synaptic plasticity.

## 5. Conclusions

Taken together, the* in vivo* expression of four reprogramming factors improved long-term memory and decreased anxiety in the animal model of chronic hypoxic-ischemic brain injury. Recovery of hippocampus-related behavior was associated with enhanced hippocampal neurogenesis and synaptic plasticity.

## Supplementary Material

Supplementary figure 1: The number of BrdU+ cells in the hippocampus two weeks after the surgical treatment in chronic hypoxic-ischemic brain injury. (A) Schematic timeline of the experiment. (B) The density of BrdU+ cells in the hippocampus was significantly higher in the treatment group than the control group (*t* = 2.349, *p* < 0.05). (C) The hippocampus of GFP control group. (D) The hippocampus of treatment group. Both C and D are images of immunohistochemistry results by confocal microscope. Cells with double positive for DAPI and BrdU are indicated with white arrows. Scale bars = 50 µm. ∗*p* < 0.05.
Supplementary figure 2: Effects of reprogramming factor expression on neurobehavioral functions in the sham-operated control group and treatment group. (A) Passive avoidance task result showed that there was no significant difference between sham-operated control group (205.7 ± 55.7 seconds) and treatment group (235.7 ± 45.7 seconds) (*n* = 6 per group). (B) Open field test result showed that there was no significant difference between sham-operated control group (9.1 ± 2.0%) and treatment group (10.3 ± 3.3%) (*n* = 6 per group).

## Figures and Tables

**Table 1:** Newly generated neurons in the hippocampus and subventricular zone after the *in vivo* expression of reprogramming factors. To identify newly generated cells, mice were injected daily with BrdU up to 12 days. Eight weeks after the treatment, histological evaluations were performed.

Group	Hippocampus			Subventricular zone		
	BrdU^+^	BrdU^+^ *β*III-tubulin^+^	BrdU^+^NeuN^+^	BrdU^+^	BrdU^+^Nestin^+^	BrdU^+^GFAP^+^
	(**×**10^3^ cells/mm^3^)	(**×**10^3^ cells/mm^3^)	(**×**10^3^ cells/mm^3^)	(**×**10^3^ cells/mm^3^)	(**×**10^3^ cells/mm^3^)	(**×**10^3^ cells/mm^3^)
GFP	8.1 ± 2.0	3.0 ± 1.6	2.1 ± 1.6	29.6 ± 7.4	3.6 ± 1.5	5.7 ± 2.0
Rep + GFP	17.6 ± 1.9^*∗*^	9.2 ± 2.3^*∗*^	13.2 ± 5.1^*∗*^	57.7 ± 4.1^*∗∗*^	15.3 ± 2.6^*∗∗*^	16.5 ± 4.8^*∗*^
Fold change	2.2	3.1	6.2	2.0	4.3	2.9

BrdU, 5-bromo-2-deoxyuridine; GFP, green fluorescent protein; Rep, reprogramming factors.

^*∗*^ *p* < 0.05, ^*∗∗*^ *p* < 0.01, and *n* = 3 per group.
