# Supplementary material for: In Vivo Expression of Reprogramming Factors Increases Hippocampal Neurogenesis and Synaptic Plasticity in Chronic Hypoxic-Ischemic Brain Injury
Source: Neural Plast. 2016 Nov 9;2016:2580837. doi: 10.1155/2016/2580837 (PMC5120183; doi:10.1155/2016/2580837)
Supplement: Supplementary file 1 — Supplementary figure 1: The number of BrdU+ cells in the hippocampus two weeks after the surgical treatment in chronic hypoxic-ischemic brain injury. (A) Schematic timeline of the experiment. (B) The density of BrdU+ cells in the hippocampus was significantly higher in the treatment group than the control group (t = 2.349, p < 0.05). (C) The hippocampus of GFP control group. (D) The hippocampus of treatment group. Both C and D are images of immunohistochemistry results by confocal microscope. Cells with double positive for DAPI and BrdU are indicated with white arrows. Scale bars = 50 µm. ∗p < 0.05. Supplementary figure 2: Effects of reprogramming factor expression on neurobehavioral functions in the sham-operated control group and treatment group. (A) Passive avoidance task result showed that there was no significant difference between sham-operated control group (205.7 ± 55.7 seconds) and treatment group (235.7 ± 45.7 seconds) (n = 6 per group). (B) Open field test result showed that there was no significant difference between sham-operated control group (9.1 ± 2.0%) and treatment group (10.3 ± 3.3%) (n = 6 per group). [file 2580837.f1.pdf]

Supplementary Figures

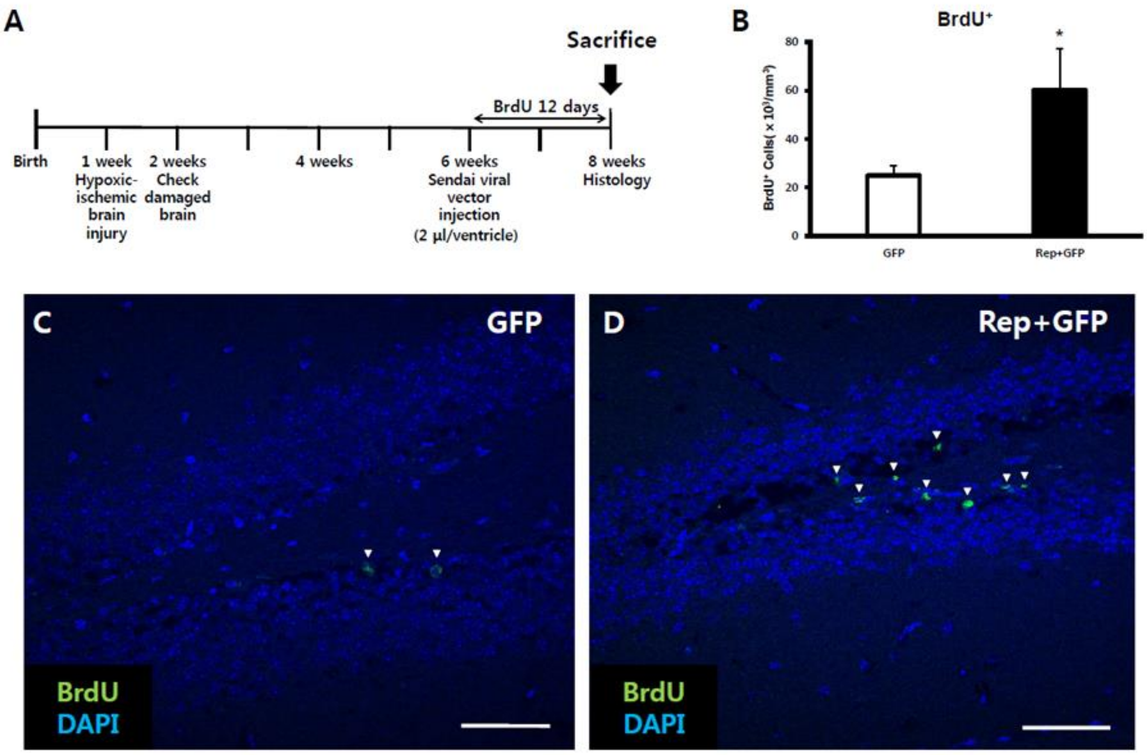

Figure S1. The number of BrdU$^+$ cells in the hippocampus two weeks after the surgical treatment in chronic hypoxic-ischemic brain injury. (A) Schematic timeline of the experiment. (B) The density of BrdU$^+$ cells in the hippocampus was significantly higher in the treatment group than the control group ($t = 2.349$, $p < 0.05$). (C) The hippocampus of GFP control group. (D) The hippocampus of treatment group. Both C and D are images of immunohistochemistry results by confocal microscope. Cells with double positive for DAPI, BrdU are indicated by the white arrow. Scale bars = 50 μm.

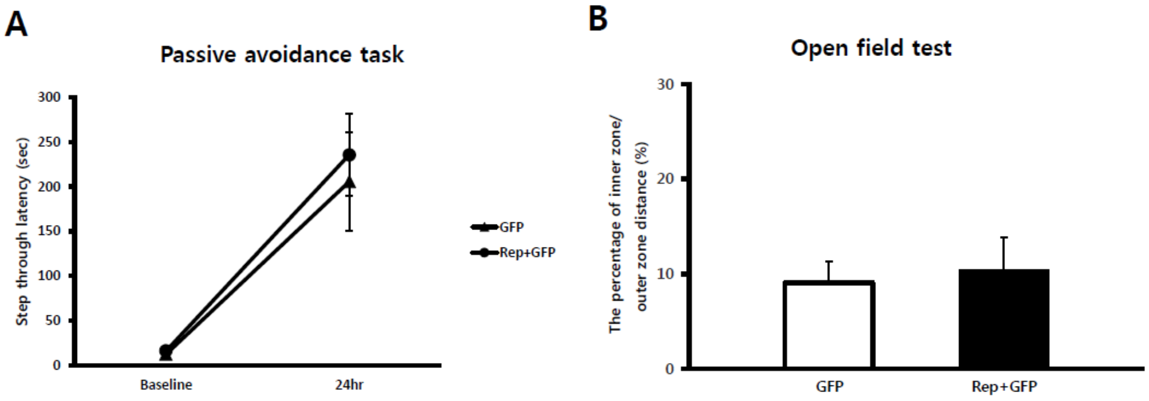

Figure S2. Effects of reprogramming factor expression on neurobehavioral functions in the sham-operated control group and treatment group. (A) Passive avoidance task result showed that there was no significant difference between sham-operated control group (205.7 ± 55.7 seconds) and treatment group (235.7 ± 45.7 seconds) (n = 6 per group). (B) Open field test result showed that there was no significant difference between sham-operated control group (9.1 ± 2.0%) and treatment group (10.3 ± 3.3%) (n = 6 per group).
